# The Effects of CYP2C19 Genotype on Proxies of SSRI Antidepressant Response in the UK Biobank

**DOI:** 10.3390/ph16091277

**Published:** 2023-09-11

**Authors:** Win Lee Edwin Wong, Chiara Fabbri, Benjamin Laplace, Danyang Li, Roos van Westrhenen, Cathryn M. Lewis, Gavin Stewart Dawe, Allan H. Young

**Affiliations:** 1 Department of Pharmacology, Yong Loo Lin School of Medicine, National University of Singapore, Singapore 117600, Singapore; 2 Healthy Longevity Translational Research Programme, Yong Loo Lin School of Medicine, National University of Singapore, Singapore 119228, Singapore; 3 Centre for Affective Disorders, Department of Psychological Medicine, Institute of Psychiatry, Psychology & Neuroscience, King’s College London, London SE5 8AG, UK; r.vanwestrhenen@psyq.nl (R.v.W.);; 4 Social, Genetic and Developmental Psychiatry Centre, Institute of Psychiatry, Psychology and Neuroscience, King’s College London, London SE5 8AF, UK; 5 Department of Biomedical and Neuromotor Sciences, University of Bologna, 40127 Bologna, Italy; 6 Psychiatry Department of Research and Innovation, Esquirol Hospital Center, 87000 Limoges, France; 7 Parnassia Psychiatric Institute/PsyQ, 1062 HN Amsterdam, The Netherlands; 8 Department of Psychiatry & Neuropsychology, Faculty of Health, Medicine and Life Sciences, Maastricht University, 6229 ER Maastricht, The Netherlands; 9 Neurobiology Programme, Life Sciences Institute, National University of Singapore, Singapore 119077, Singapore; 10 Department of Medical and Molecular Genetics, Faculty of Life Sciences and Medicine, King’s College London, London WC2R 2LS, UK; 11 South London & Maudsley NHS Foundation Trust, Bethlem Royal Hospital, Monks Orchard Road, London BR3 3BX, UK

**Keywords:** pharmacogenetics, cytochrome P450, treatment response, antidepressants

## Abstract

Selective serotonin reuptake inhibitors (SSRIs) are the most commonly used psychopharmaceutical treatment for major depressive disorder (MDD), but individual responses to SSRIs vary greatly. CYP2C19 is a key enzyme involved in the metabolism of several drugs, including SSRIs. Variations in the CYP2C19 gene are associated with differential metabolic activity, and thus differential SSRI exposure; accordingly, the CYP2C19 genotype may affect the therapeutic response and clinical outcomes, though existing evidence of this link is not entirely consistent. Therefore, we analysed data from the UK Biobank, a large, deeply phenotyped prospective study, to investigate the effects of CYP2C19 metaboliser phenotypes on several clinical outcomes derived from primary care records, including multiple measures of antidepressant switching, discontinuation, duration, and side effects. In this dataset, 24,729 individuals were prescribed citalopram, 3012 individuals were prescribed escitalopram, and 12,544 individuals were prescribed sertraline. Consistent with pharmacological expectations, CYP2C19 poor metabolisers on escitalopram were more likely to switch antidepressants, have side effects following first prescription, and be on escitalopram for a shorter duration compared to normal metabolisers. CYP2C19 poor and intermediate metabolisers on citalopram also exhibited increased odds of discontinuation and shorter durations relative to normal metabolisers. Generally, no associations were found between metabolic phenotypes and proxies of response to sertraline. Sensitivity analyses in a depression subgroup and metabolic activity scores corroborated results from the primary analysis. In summary, our findings suggest that CYP2C19 genotypes, and thus metabolic phenotypes, may have utility in determining clinical responses to SSRIs, particularly escitalopram and citalopram, though further investigation of such a relationship is warranted.

## 1. Introduction

Major depressive disorder (MDD) is a mood disorder that affects an estimated 246 million people globally [1], imposing significant societal burden, hindering daily living, straining the healthcare system, and reducing work productivity [2,3]. While numerous forms of treatment are available to address MDD, pharmacotherapy with antidepressants remains the most frequently administered option for moderate to severe forms of depression [4,5]. Among the classes of antidepressants available, selective serotonin reuptake inhibitors (SSRIs) have been a standard treatment option for several decades, a status attributable to the substantial evidence base that supports their effectiveness and safety [6,7].

However, despite the widespread use of antidepressants, among them SSRIs, individual responses to this pharmacotherapy vary significantly, with a large proportion of antidepressant-treated individuals experiencing therapeutic failure [8,9]; these cases are characterised by a lack of response to medication or adverse side effects. Several factors contribute to the variability in response to antidepressants. For example, the diagnostic heterogeneity of affective disorders [10] means that individuals with a range of comorbidities are often grouped into the same category, such that differences in response are inevitable. Genetic variations have also been shown to mediate differential antidepressant responses [11,12], with variations in the genes that encode for the Cytochrome P450 (CYP) superfamily of proteins linked to the response to medication [13].

The CYP2C19 enzyme in particular is responsible for the metabolism of many compounds, including the SSRIs citalopram, escitalopram, and sertraline, among other drugs [14]. The metabolic capacity of CYP2C19 is determined by its corresponding allelic makeup, with patients classified into different metaboliser status categories according to their genotypes and enzymatic capacity [15,16]. There is strong clinical evidence corroborating associations between the CYP2C19 metaboliser status and antidepressant exposure, particularly for citalopram, escitalopram, and sertraline [17,18,19]. Poor and intermediate metabolisers exhibit higher drug exposure, as they metabolise slower, while ultrarapid metabolisers exhibit lower drug exposure, as they metabolise much faster. Accordingly, the Dutch Pharmacogenetics Working Group (DPWG) guidelines have suggested that CYP2C19 genotyping may be beneficial before the start of administering citalopram, escitalopram, and sertraline for predicting their therapeutic efficacy [20,21,22]. The Clinical Pharmacogenetics Implementation Consortium (CPIC) further supports these recommendations, suggesting dose adjustments corresponding to the metaboliser status for these medications [23].

While the effects of genetic variation in CYP2C19 on SSRI metabolism are well-established, there remains inconsistency across studies exploring associations between CYP2C19 genotypes and clinical indications of the response to SSRIs. Some studies have reported associations between CYP2C19 variants and endpoints that reflect antidepressant response efficacy [18,24], while other studies have found no such relationship [25,26]. The divergency in these reports is likely due to several factors, including the relatively small sample sizes used in certain studies, different study designs, and the difficulty of measuring the treatment response in clinical settings—the outcomes range from switching the antidepressant medication to self-reported measures of tolerability and side effects. As such, additional research is clearly necessary to elucidate the putative relationships between the CYP2C19 genotype and SSRI response.

Therefore, our study aims to evaluate associations between CYP2C19 genotypes (and concomitant metaboliser phenotypes) and several proxies of the treatment response for citalopram, escitalopram, and sertraline. We conducted these analyses using the UK Biobank [27], a large, deeply phenotyped, health study of 500,000 individuals. This resource includes genetic data that enable us to determine the CYP2C19 metaboliser status, as well as primary care records for a subset of 222,054 participants. These records contain information on clinical events and prescriptions at the primary care level, allowing us to monitor prescription patterns for our SSRIs of interest. Consequently, we investigated proxies of SSRI response using outcomes derived from these records, including antidepressant switching, discontinuation, duration, and side effects.

## 2. Results

### 2.1. Study Demographics and CYP2C19 Metaboliser Phenotype Prevalence

In total, there were 33,094 individuals in our dataset that had been prescribed any of escitalopram, citalopram, or sertraline at least once, and had a known metaboliser status. The average age across this dataset was 55.2 years (S.D. = 8.1), and 66.2% of the sample was female (n = 21,890). The majority of participants were classified as CYP2C19 normal metabolisers based on their called genotypes (n = 13,098, 39.6%), while the least common metaboliser classification was the poor metaboliser phenotype (n = 905, 2.7%). The remainder of the sample comprised fast metabolisers (n = 10,444, 31.6%) and intermediate metabolisers (n = 8619, 26.0%). In this sample, the prevalences of rapid and ultrarapid metabolisers, which comprised our “fast” metaboliser phenotype were 26.9% and 4.7% respectively, which was consistent with prior prevalence estimates [28]. Among our three SSRIs of interest, citalopram was the most prescribed (n = 24,729), followed by sertraline (n = 12,544), and then escitalopram (n = 3012). The distribution of metaboliser status and SSRI prescription, as well as basic demographic information, are presented in Table 1.

### 2.2. Associations between Metaboliser Phenotype and Antidepressant Switching

**Figure 1:**
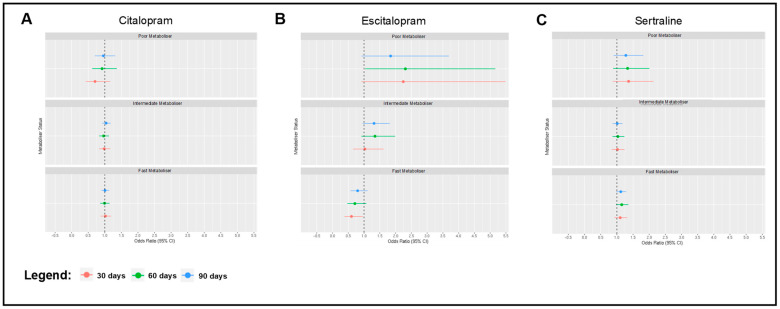
Forest plots that highlight odds ratios and 95% confidence intervals for regression models evaluating associations between CYP2C19 metaboliser status and SSRI switching within 30 days (red), 60 days (green), and 90 days (blue), with normal metabolisers as the reference group. Models were adjusted for age at first prescription, sex, genotype batch, and binary overlap with an antipsychotic prescription. Associations are shown for switching from (**A**) citalopram, (**B**) escitalopram, and (**C**) sertraline to another antidepressant.

We assessed whether there were any associations between the CYP2C19 metaboliser status and early antidepressant switching at 30 days, 60 days, and 90 days (Figure 1). Poor metabolisers on escitalopram were more likely to switch to another antidepressant, relative to normal metabolisers, at 60 days (OR = 2.311, 95% CI = 1.034–5.163, *p* = 0.041). However, fast metabolisers on escitalopram were less likely to switch, compared to normal metabolisers, at 30 days (OR = 0.601, CI = 0.374–0.966, *p* = 0.036). The effect size of these associations at other timepoints were similar, but did not reach statistical significance (see Supplementary Table S1). There were no significant associations pertinent to switching for citalopram and sertraline.

### 2.3. Associations between Metaboliser Phenotype and Antidepressant Discontinuation

**Figure 2:**
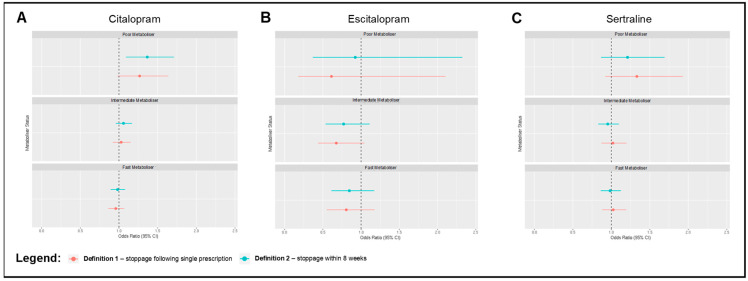
Forest plots that highlight odds ratios and 95% confidence intervals for regression models evaluating associations between CYP2C19 metaboliser status and SSRI discontinuation defined in two ways, with normal metabolisers as the reference group. Definition 1 (red) entails abrupt stoppage of prescriptions following a single prescription, while definition 2 (blue) entails abrupt stoppage following a prescription window lasting less than 8 weeks. Models were adjusted for age at first prescription, sex, genotype batch, and binary overlap with an antipsychotic prescription. Associations are shown for discontinuation of (**A**) citalopram, (**B**) escitalopram, and (**C**) sertraline.

We also tested the effect of CYP2C19 metaboliser status on antidepressant discontinuation (Figure 2). Compared to normal metabolisers, poor metabolisers who were prescribed citalopram were more likely to discontinue antidepressant usage, as defined by a stoppage following a brief prescription period (OR = 1.366, CI = 1.090–1.712, *p* = 0.007). No other significant associations between the metaboliser phenotypes and our two definitions of antidepressant discontinuation were identified across all three SSRIs of interest (Supplementary Table S2).

### 2.4. Associations between Metaboliser Phenotype and Antidepressant Duration

Next, we evaluated associations between the metaboliser status and the natural logarithmic transformation of antidepressant duration, as defined on the basis of the number of prescriptions (“count-based definition”) and total weeks of prescription (“weeks-based definition”); the results are presented in Table 2. Among the individuals prescribed escitalopram, poor metabolisers used escitalopram for a shorter duration compared to normal metabolisers, for both our count-based definition (β = −0.351, SE = 0.174, *p* = 0.043) and our total weeks definition (β = −0.369, SE = 0.185, *p* = 0.046). Within the cohort of individuals prescribed citalopram, the intermediate metabolisers had a shorter duration of citalopram use compared to normal metabolisers, demonstrated using both the count-based (β = −0.048, SE = 0.024, *p* = 0.045) and total weeks (β = −0.051, SE = 0.025, *p* = 0.043) definitions. Among the individuals prescribed sertraline, fast metabolisers were on sertraline for a shorter duration than normal metabolisers, though associations were only significant for our total weeks definition (β = −0.071, SE = 0.032, *p* = 0.029).

### 2.5. Associations between Metaboliser Phenotype and Side Effects

**Figure 3:**
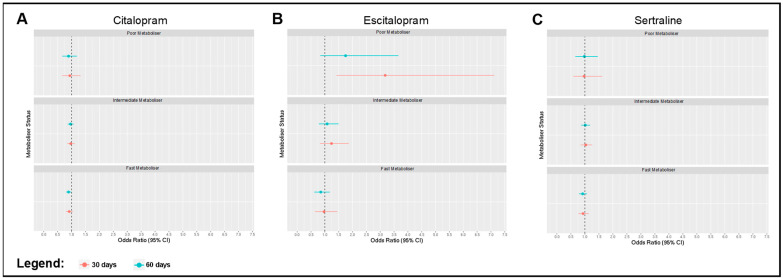
Forest plots depicting odds ratios and 95% confidence intervals for regression models evaluating associations between CYP2C19 metaboliser phenotype and the presence of side effects within 30 days (red) and 60 days (blue) of first prescription, with normal metabolisers as the reference group. Models were adjusted for age at first prescription, sex, genotype batch, and binary overlap with an antipsychotic prescription. Associations are shown for side effects following (**A**) citalopram, (**B**) escitalopram, and (**C**) sertraline prescription.

Finally, we investigated associations between the metaboliser status and a measure of psychotropic side effects, derived using primary care clinical event records (Figure 3). For individuals prescribed escitalopram, poor metabolisers were more likely to have side effects within 30 days of the first prescription, relative to normal metabolisers (OR = 3.179, CI = 1.420–7.121, *p* = 0.005), which was consistent with poorer metabolic capacity. This effect reduced substantially when extending the side effect window to 60 days, and was not statistically significant (OR = 1.748, CI = 0.836–3.656, *p* = 0.138). For individuals prescribed citalopram, fast metabolisers (that metabolise quicker) were less likely to have side effects within 60 days of the first prescription versus normal metabolisers (OR = 0.892, CI = 0.801–0.995, *p* = 0.040), though this relationship was not significant at 30 days (OR = 0.917, CI = 0.803–1.046, *p* = 0.198). There were no other significant associations; the results from all of the regression tests are presented in Supplementary Table S3.

### 2.6. Sensitivity Analyses

We also conducted two sensitivity analyses to assess the robustness of our findings. In the first analysis, we repeated our regression tests in a subset of participants that were ascertained to have a “broad depression” phenotype. Overall, 78.5% of the individuals prescribed citalopram, 89.1% of individuals prescribed escitalopram, and 79.0% of individuals prescribed sertraline were categorised as having broad depression. The results of association testing in this subgroup are presented in Supplementary Tables S4–S7.

Nearly all effect sizes identified during sensitivity analysis with the broad depression subgroup were consistent with those from the primary analysis: poor metabolisers on escitalopram remained more likely to switch antidepressants at 60 days (OR = 2.371, CI = 1.017–5.530, *p* = 0.046), and stayed on escitalopram for a shorter duration than normal metabolisers, while poor metabolisers on citalopram remained more likely to discontinue usage (OR = 1.426, CI = 1.073–1.896, *p* = 0.015); in this instance, an association between poor metaboliser status and citalopram discontinuation was also present for our other definition, which was predicated on number of prescriptions (OR = 1.446, CI = 1.054–1.983, *p* = 0.022). The associations between intermediate metaboliser status and citalopram duration (count-based definition, β = −0.055, SE = 0.027, *p* = 0.043), as well as between fast metaboliser status and sertraline duration (weeks-based definition, β = −0.082, SE = 0.037, *p* = 0.026), also remained statistically significant, with effect sizes similar to those derived in the primary analysis, as did the relationship between fast metaboliser status and reduced likelihood of side effects following citalopram prescription (OR = 0.881, CI = 0.780–0.995, *p* = 0.041). Furthermore, new associations were identified in the broad depression subgroup, with fast metabolisers on escitalopram more likely to discontinue usage compared to normal metabolisers (OR = 0.655, CI = 0.441–0.973, *p* = 0.036).

However, certain effect sizes attenuated when we limited the analysis to individuals with broad depression. Fast metabolisers on escitalopram were no longer less likely to switch than normal metabolisers (OR = 0.890, CI = 0.775–1.022, *p* = 0.097). Moreover, poor metabolisers prescribed escitalopram were no longer more likely to have side effects following the first prescription (OR = 1.737, CI = 0.629–4.798, *p* = 0.287).

In our second sensitivity analysis, we repeated association testing using the CYP2C19 activity score as a predictor in lieu of categorical metaboliser phenotypes. For individuals prescribed escitalopram, a higher CYP2C19 activity score was associated with reduced odds of switching to another antidepressant within 30 days (β = −0.311, SE = 0.110, *p* = 0.005), 60 days (β = −0.337, SE = 0.096, *p* < 0.001), and 90 days (β = −0.249, SE = 0.079, *p* = 0.002). Furthermore, a higher activity score was linked to a longer duration on escitalopram for both the count-based definition (β = 0.072, SE = 0.031, *p* = 0.021) and the weeks-based definition (β = 0.068, SE = 0.031, *p* = 0.030). Among the individuals prescribed citalopram, a higher activity score was associated with decreased odds of discontinuation for the definition based on the prescription period (β = −0.051, SE = 0.023, *p* = 0.029). There were no significant associations between the CYP2C19 activity score and the likelihood of side effects following the first SSRI prescription; the full association testing results are presented in Supplementary Tables S8–S11.

## 3. Discussion

In this study, we used the large UK Biobank resource to link CYP2C19 metabolic capacity with several proxy measures of antidepressant response. Our findings showed several associations between CYP2C19 metaboliser phenotypes and differential response to SSRIs, particularly for escitalopram and citalopram (es/citalopram); these relationships were demonstrated in several multiple regression models where either the metaboliser status or the metabolic activity score were used as predictors. This strengthens the argument that early CYP2C19 genotyping prior to prescription may have value in improving the therapeutic efficacy of SSRIs, an important implication for pharmacogenetics.

Our findings were generally consistent with the pharmacological rationale outlined in the dosing recommendation guidelines from the CPIC [23] and DWPG [20], as well as the Dutch guideline on the implementation of pharmacogenetics for psychiatrists [22]. Both guidelines advise a reduction in the starting dose for CYP2C19 poor metabolisers prescribed escitalopram, a recommendation rooted in the wealth of evidence reporting a link between poor metaboliser status (i.e., the absence of CYP2C19 enzymatic activity) and significantly higher escitalopram plasma concentrations [18,29,30]. Thus, our findings can be explained by this well-established genotype effect on exposure, with poor metabolisers having higher odds of escitalopram switching and side effects, as well as a shorter average duration on the antidepressant, compared to normal metabolisers. In addition, the correlation between decreasing metabolic capacity and increased citalopram serum exposure [31,32], as reflected in the DPWG recommendation, supports the increased odds of discontinuation and a shortened duration on citalopram that poor metabolisers and intermediate metabolisers demonstrated in our study, respectively. As expected, given the rapid clearance that is concomitant with higher metabolic capacity, citalopram fast metabolisers were less likely to display side effects following the first prescription.

In addition to the aforementioned research focused on plasma SSRI concentration, there is a substantial body of literature reporting on the putative relationship between the CYP2C19 metaboliser status and SSRI therapeutic response, though the evidence is more ambiguous with conflicting reports. Nonetheless, our findings are supported by several of these studies across different measures of treatment efficacy. In the only other study that investigated antidepressant switching as a proxy of therapeutic failure, Jukic et al. reported that poor metabolisers switched from escitalopram 3.3 times more frequently than did normal metabolisers [18], a finding that our results directly support, despite the differences in our time window for switching (we used 30, 60, and 90 days instead of 1 year). In a cohort of adolescents from the Cincinnati Children’s Hospital Medical Center, poor metabolisers and intermediate metabolisers were more likely to discontinue es/citalopram treatment than normal metabolisers [33]. We found the same relationship between poor metabolisers and citalopram discontinuation in our sample, as well as in the direct association we identified between the CYP2C19 activity score and escitalopram duration. Several studies also explored CYP2C19 genotypes in the context of changes in the Hamilton Depression Rating (HAMD) Scale [34] as a measure of therapeutic efficacy. In one small study, intermediate metabolisers on citalopram exhibited significantly higher HAMD scale scores 8 weeks after baseline, which was indicative of lower therapeutic efficacy [35]. Jokovic et al. reinforced this finding, demonstrating that “slow metabolisers” (a composite of poor and intermediate metabolisers) had a significantly lower reduction in their HAMD scores during their study [36]. Our findings that linked intermediate metabolisers to shorter citalopram durations are in line with these reports, though our outcomes were only proxies for efficacy rather than direct measures of self-reported efficacy, as used in aforementioned studies (self-report efficacy responses were not available for our analysis). A putative relationship between the CYP2C19 genotypes and SSRI response efficacy is further supported by several clinical trials [37,38,39,40,41,42], where the use of pharmacogenetic testing in guiding treatment improved patient antidepressant response, as assessed on a range of different scales.

Several studies have also previously investigated the effects of CYP2C19 variations on side effects, measured in a variety of ways. For example, in the study from Jokovic et al. [36], slow metabolisers had a higher average intensity score on the Toronto Side Effects Scale, which is indicative of lower tolerability in this group, relative to normal metabolisers. A meta-analysis from Fabbri et al. [24] summarises the evidence well: poor metabolisers showed an increased risk of gastro-intestinal, sexual, and central nervous side effects at weeks 2–4 following baseline. We corroborate this conclusion, demonstrating that poor metabolisers are more likely to have side effects within 4 weeks, where our definition of side effects based on the UKU-SERS encompassed all subclasses mentioned above. While these results are concordant, it is worth highlighting that we used a different definition of side effects, leveraging primary care clinical records rather than any rating scale covered in the meta-analysis. Our approach exhibits greater similarity to the methods Aldrich et al. used in the Cincinnati children cohort [33], where natural language processing was applied to analyse electronic medical records. They identified that poor metabolisers had the highest number of side effects experienced, while ultrarapid metabolisers had the lowest total side effects, following es/citalopram prescription; this directly parallels our own analyses. While the use of electronic records to derive adverse drug reactions has certain limitations, this approach has been previously adopted and shown to be rigorous [43,44,45].

While our results largely support existing evidence in this domain, it is also important to acknowledge where our findings were not consistent within the context of our study, and in the context of other reports. For example, while we identified an association between escitalopram poor metabolisers and early switching at 60 days, this was not strictly replicated at 30 days or 90 days. While these associations did not reach statistical significance, this can be attributed to an underpowered analysis due to the small number of poor metabolisers on escitalopram, as well as the consistently high effect sizes across all timepoints. In addition, discrepancies between the specific outcome definitions were sporadically identified; for example, citalopram poor metabolisers were linked to one definition of discontinuation but not another. Notably, however, the association with the other discontinuation definition was close to the significance threshold (*p* = 0.072). Beyond some degree of inconsistency between the outcome definitions, our findings also conflict with some prior observations. Jukic et al. [18] reported an association between ultrarapid metaboliser status and increased odds of switching, as a proxy of therapeutic failure, consistent with the rationale that quicker clearance would mean poorer efficacy, and thus higher odds of switching. However, we uncovered the opposite relationship in our study, with fast metabolisers on escitalopram switching less than normal metabolisers. These differences could have arisen from the subtle differences in the switch definition, the different switch time windows employed (1–3 months versus 1 year), or the relatively small sample sizes in both studies, possibly underpowering our analyses. Two studies in the Sequenced Treatment Alternatives to Relieve Depression sample (STAR*D) also failed to identify any consistent associations between CYP2C19 genetic variations and clinical response or tolerance [25,26], which contradicts our findings to an extent. This disparity is possibly due to the different nature of outcome measures for response, with the use of self-report and study adherence data in STAR*D compared to our use of primary care records to identify prescription patterns. Campos et al. [46] also reported a link between intermediate metabolisers on sertraline and the likelihood of side effects; we uncovered no such association, though once again, there was variation in how we defined the side effect variable.

Nonetheless, we consider our results to be relatively robust, particularly as our sensitivity analyses reinforced our primary conclusions. In both our subgroup analysis and our analyses using the activity score, most associations identified in the main analyses remained significant, suggesting that any effects of CYP2C19 genotypes on the responses to citalopram, escitalopram, and sertraline are linked to metabolic capacity and are independent of depression status. However, it is also important that our results are interpreted with caution. We presented results in this study without adopting any corrections for multiple testing burden, as we intended to capture all possible associations, and as there is no clear consensus on the appropriate method to use in this context [47,48], particularly given the multiple measures of a broad treatment response outcome; however, without any corrections for multiplicity, it is difficult to rule out false positives. For example, if a Bonferroni correction was applied for the four outcomes [49], only some of the associations (particularly for citalopram duration and discontinuation, and escitalopram side effects) would remain statistically significant against the adjusted threshold. Nevertheless, the large effect sizes and consistency across results suggest the presence of some association between CYP2C19 phenotypes and SSRI response, though we are cognisant to emphasise care and context when interpreting these results.

Our study yields insights into clinical guidelines and personalized treatment approaches in the context of SSRIs. The DWPG and CPIC advise against prescribing escitalopram to ultrarapid metabolisers [20,50]; however, our data indicate that fast metabolisers, characterised by their heightened metabolic capacity, exhibit a reduced likelihood of switching medications while on escitalopram compared to normal metabolisers. While our findings offer a valuable contradictory perspective, they should be considered within the broader clinical context. We were unable to ascertain whether the escitalopram dosages in our sample differed from those in previous studies, and the heterogeneity in the body of evidence on the link between ultrarapid metabolisers and escitalopram therapeutic response underscores the complexity of this relationship. With regards to CYP2C19 poor metabolisers, our study reinforces existing evidence [18] that has informed the development of the DPWG and CPIC guidelines: our results suggest that adjusting the initial dose of escitalopram represents a viable clinical strategy for individuals in this category. In terms of practical implications for real-world prescription practices, our findings lend credence to the potential value of preemptive pharmacogenetic testing. However, it is crucial to recognize that the clinical integration of preemptive testing faces multifaceted challenges extending beyond established scientific evidence. Notably, our study could not account for the complexities of medication combinations and comorbid conditions in real-world settings. Additionally, the cost-effectiveness of preemptive testing remains a matter of ongoing investigation, particularly given the heterogeneity of existing pharmacogenomic testing panels [21]. Nonetheless, our findings support a broader shift towards personalised treatments, a paradigm shift where patients may benefit from reduced susceptibility to side effects and active participation in shared decision-making with healthcare providers for the optimal treatment course. While the practical integration of pharmacogenetic testing into routine patient care continues to evolve, our findings solidify the scientific foundation underlying this shift.

This study has several strengths that support its reliability. Firstly, this study was relatively well-powered compared to several previous investigations, with a fairly large number of participants and longitudinal prescription information. In addition, we employed a comprehensive approach to defining the outcomes, deriving these fields in multiple ways and at multiple timepoints to better capture the complexity of the clinical antidepressant response. Given the constraints with UK Biobank phenotyping, we utilised primary care records in this study, avoiding the use of self-reported scales and questionnaires in determining treatment response. Consequently, this study circumvented some of the biases (e.g., response and rater bias) that are inherent to these measurements [51,52]. Finally, we conducted two sensitivity analyses, which yielded results that supported the associations identified in the primary analyses, thereby strengthening the study’s robustness and ensuring greater confidence in the accuracy of our conclusions.

There were, however, also several limitations in this study worth highlighting. First, our outcome measures were derived based on prescription patterns from primary care records, such that they do not directly reflect SSRI response or tolerance; moreover, there was no way to verify that a prescription implied consumption of a given antidepressant. The retrospective nature of this analysis placed constraints on deriving relevant outcomes to the SSRI response, and hence on our usage of several prescription-based outcomes and definitions to capture overall response. Another limitation was the relatively small number of escitalopram users in the UK Biobank, though this sample size was comparable with other investigations to date [18,46]. Moreover, the broad depression phenotype used in our subgroup analyses was relatively lenient and based on self-reported information rather than a structured diagnostic assessment [53]; as such, this phenotype is likely to capture a wider range of individuals than classic definitions of depression. In addition, this definition was derived from data recorded at baseline assessment such that it could not be synchronised with the prescription timeframes, further limiting interpretation of this phenotype. However, other stringent definitions previously used in the UK Biobank were only available for a subset of participants who completed an online follow-up questionnaire [54], which would heavily reduce our sample size, rendering the broad phenotype most suitable for a sensitivity analysis. In addition, it was not within the scope of this study to analyse the possible complex confounding drug–drug interactions with SSRIs [55,56], such that we were not able to capture the effects that any concomitant drugs may have on the metabolism or proxy outcomes of our SSRIs of interest. In a similar vein, we did not explore the presence of adjunct or augmentation therapy with other medications, inferring that this potential proxy of response from prescription records also fell beyond the scope of this investigation. Finally, the SSRI dosage information could provide additional contexts to aid the interpretation of our analyses, particularly for attenuated effect sizes in some of our sensitivity analyses; however, the dosages were not readily available in the UK Biobank, and it was beyond the study’s scope to derive this information.

In summary, while more research must be carried out to fully ascertain the relationship between CYP2C19 metaboliser phenotypes and SSRI therapeutic efficacy, our findings suggest that reduced metabolic capacity may be linked to an increased likelihood of switching, discontinuation, and side effects, as well as shorter durations, particularly for es/citalopram. These conclusions should be interpreted alongside a demonstrated lack of associations between CYP2C19 genotypes and proxies of therapeutic response to sertraline in the UK Biobank, contrasting with prior findings. Nonetheless, taken altogether, these results imply that CYP2C19 genotyping should at least be considered in people experiencing problems with es/citalopram, such as side effects or lack of efficacy. These findings also suggest that pre-emptive genotyping may be useful in informing clinical decision-making for these SSRIs.

## 4. Methods

### 4.1. Study Population

The study data were obtained from the UK Biobank, a health study comprising around 500,000 participants from different regions of the United Kingdom aged 40–70 years at their baseline assessment [27]. The initial recruitment was finished in 2010, following over 9 million postal invitations to participate—subsets of participants completed repeated measurements as well as new measurements in the ensuing years. Genotyping data were available for the majority (approximately 488,000) of these individuals, with DNA samples extracted from whole blood and genotyped using either the Affymetrix UK Biobank Axiom Array or the Affymetrix UK BiLEVE Axiom Array [57]. The two genotype arrays used are very similar, with over 95% overlap in their SNP marker content [58], minimizing any potential variability. Genotype imputation was subsequently performed using IMPUTE4 software and the Haplotype Reference Consortium (HRC) data as the primary reference panel [59]. The UK Biobank also provides a wide range of phenotypic data, obtained from physical assessments, biological samples, hospital inpatient records, and touchscreen questionnaires. In addition, the UK Biobank contains a comprehensive dataset of primary care events from various healthcare providers across England, Wales, and Scotland, including records on registrations, clinical events, and prescriptions [60], which are available for approximately 230,000 participants. We used this extensive collection of data to create several phenotype definitions, as described in more detail below.

### 4.2. CYP2C19 Genotyping and Metaboliser Phenotype

The CYP2C19 genotypes and metaboliser statuses were obtained using UK Biobank return 3388 from McInnes et al. [61], which consists of called pharmacogenetic star alleles and metaboliser phenotypes derived using a Python program named PGxPOP. In brief, PGxPOP quickly identifies and reports pharmacogenetic star alleles (popular nomenclature that corresponds to functional haplotype patterns which influence drug metabolism) and haplotypes from phased multisample VCFs, based on PharmCAT allele definition files [62], which comprehensively cover CYP2C19 genetic variants. PGxPOP then uses guidelines from the DPWG and CPIC to match these haplotypes to predicted metabolic phenotypes. Thus, the individuals in this study were grouped into 4 categories based on predicted CYP2C19 phenotypes, which in increasing order of metabolic capacity are the following: poor metabolisers, intermediate metabolisers, normal metabolisers, and fast metabolisers. Certain groups specify separate rapid and ultrarapid metaboliser groups [46,63], corresponding to individuals with one increased function allele and one normal function allele. However, in this study, we combined the rapid and ultrarapid metaboliser phenotypes returned by PGxPOP into a single category (“fast” metabolisers). Prior research has found that the rapid and ultrarapid metaboliser phenotypes exhibited similar pharmacokinetic properties, specifically with comparable escitalopram serum concentrations following intake [18,36]; consequently, we combined the two groups to bolster the statistical power in uncovering any associations that were consistent with previous research with CYP2C19 and clinical outcomes [36]. Full information about the matching between allele, haplotype, and phenotype can be found in previously published articles [43,61].

Individuals whose predicted metaboliser phenotype was classified as “indeterminate” or “not available” were excluded from the analyses. These classifications correspond to individuals with star alleles that have an unknown or uncertain function, and those whose alleles did not align precisely with any star allele (preventing the assignment of a phenotype), respectively.

### 4.3. Outcomes

Primary care records in the UK Biobank were collected through linkages with various data suppliers, including EMIS and Vision and TPP SystmOne; they were organised into three different tables: registration records, primary care clinical events (for example, consultations, diagnoses, symptoms), and prescription data (coded using the read v2, BNF, and dm+d coding systems). The linked primary care data were available only in a subset of UK Biobank individuals (n = 222,054). These records were used to derive several study outcomes that were used as proxies of the drug response for our SSRIs of interest. Outcomes were created for each of citalopram, escitalopram, and sertraline, and included switching, discontinuation, duration, and a measure of side effects.

Antidepressant switching was defined as a switch from a given SSRI (citalopram, escitalopram, or sertraline) to another antidepressant within 30 days, 60 days, or 90 days from the last prescription of said SSRI. At 30 days and 60 days, our definition of switching allowed for a maximum of 1 prescription before the switching date, and 1 prescription after the switching date; at 90 days, our definition was less stringent, allowing for a maximum of 2 prescriptions before and after the switching date. The controls for antidepressant switching had to have at least 3 consecutive prescriptions of a given SSRI, with no record of any previous switch from that SSRI.

Antidepressant discontinuation was defined in two distinct ways, based on prescription records in the UK Biobank. Our first definition required a complete stoppage of antidepressant prescriptions following a single prescription of a given SSRI, precluding cases where the given SSRI was prescribed multiple times. Our second definition required an abrupt stoppage of antidepressant prescriptions following a prescription period lasting less than 8 weeks for a given SSRI, irrespective of the number of prescriptions during the 8 weeks. The controls were defined in the same manner as they were with antidepressant switching (at least 3 consecutive prescriptions of a given SSRI).

The antidepressant duration was measured in two ways. Our first definition for the duration was a count of the total number of prescriptions an individual was prescribed across their primary care records for a given SSRI. Our second definition for the duration was based on the total number of weeks an individual was prescribed an SSRI of interest across all prescription windows. The prescription windows were firstly defined as periods with less than 90 days between consecutive prescriptions, after which the time elapsed in weeks within each window was calculated. The total number of weeks across all of the prescription windows was then calculated and used as a measure of the duration for each SSRI.

Finally, we defined adverse side effects using both prescription records and primary care clinical event records. Using the UK Biobank clinical coding lookup maps, we identified all primary care clinical event codes (read v2 and CTV3) that were pertinent to side effects for psychotropic drugs, based on the UKU side effect rating scale (UKU-SERS), a comprehensive rating scale [64]. A full list of the 1220 clinical event codes that are pertinent to the UKU-SERS can be found in Supplementary Table S12. The dates of these clinical events were then aligned with dates of prescription records, such that the presence of a side effect was defined as the occurrence of a pertinent clinical event within a short timeframe (30 days or 60 days) after first prescription for each SSRI of interest (citalopram, escitalopram, or sertraline). In addition, any pertinent clinical event was only considered a side effect if it did not also occur within the 30 days prior to the first prescription, in order to ensure the clinical event was not simply a follow-up visit for a condition existing prior to SSRI prescription.

### 4.4. Statistical Analyses

All statistical analyses were performed using R version 4.1.1 [65] in RStudio [66], on the King’s Computational Research, Engineering and Technology Environment [67]. Our code made use of the ukbkings [68,69], dplyr [70], stringr [71], and ggplot2 [72] R packages.

Logistic regressions were conducted to evaluate any associations between CYP2C19 metaboliser phenotypes (with normal metabolisers as a reference category) and binary outcomes, including antidepressant switching, discontinuation, and incidences of any side effects. We adjusted for age at the recorded first prescription (of a given SSRI), for sex, and the genotyping batch. Linear regression models were used to assess associations between CYP2C19 metaboliser phenotypes (with normal metabolisers again being the reference category) and the natural logarithmic transformation of the antidepressant duration, adjusting similarly for the age at first prescription, sex, and genotyping batch. In order to comprehensively capture all of the potential associations within the broad theme of SSRI response, no correction for multiple testing was performed.

### 4.5. Sensitivity Analyses

We aimed to assess whether associations would change if the analyses were limited to those individuals with depression. Consequently, all of the regression analyses were repeated in a subgroup of patients with “broad depression”, a tractable UK Biobank phenotype for depression that has previously been defined by Howard et al. [53]. In brief, broad depression caseness was defined via touchscreen responses to questions that asked participants about help-seeking behaviour with general practitioners and psychiatrists. While depression has been defined in several ways in the UK Biobank, the broad depression phenotype provides the greatest number of cases, and had the highest genetic correlation with clinically defined MDD. In addition, we conducted a second set of sensitivity analyses using CYP2C19 activity score as a predictor rather than the categorical metabolic phenotypes in our regression models. The CYP2C19 activity value was calculated by assigning each star allele a score between 0 and 2, based on its effect on metabolic activity, where the activity score was doubled if alleles were duplicated [25,73,74]. The complete assignment of star alleles to the activity score is presented in Supplementary Table S13.

## Figures and Tables

**Table 1:** Study demographic information and distribution of CYP2C19 metaboliser phenotypes and SSRI prescriptions.

	Citalopram	Escitalopram	Sertraline	Total Sample
**Metaboliser phenotype**				
Poor, N (%)	675 (2.7%)	75 (2.5%)	349 (2.8%)	905 (2.7%)
Intermediate, N (%)	6468 (26.2%)	793 (26.3%)	3306 (26.4%)	8619 (26.0%)
Normal, N (%)	9731 (39.3%)	1197 (39.7%)	4965 (39.6%)	13,098 (39.6%)
Fast, N (%)	7855 (31.7%)	947 (31.4%)	3924 (31.3%)	10,444 (31.6%)
**Total number of individuals prescribed SSRI**				
N	24,729	3012	12,544	33,094
**Demographics**				
Age (mean ± SD)	55.0 (8.1)	55.2 (7.9)	55.4 (8.2)	55.2 ± 8.1
Sex (% Female)	66.9%	68.1%	65.6%	66.2%

**Table 2:** Associations between CYP2C19 metaboliser status and antidepressant duration, defined using a count-based definition and a weeks-based definition. The count-based definition corresponds to the total number of prescriptions an individual had across primary care records for a given SSRI. The weeks-based definition corresponds to the total number of weeks an individual was prescribed a given SSRI across all prescription windows in their primary care records. The mean and standard deviation of antidepressant duration is also presented according to metaboliser status for each antidepressant.

Citalopram						
Metaboliser Phenotype	Count-Based Definition			Weeks-Based Definition		
	Mean (SD)	β (SE)	*p*	Mean (SD)	β (SE)	*p*
Poor metaboliser	21.3 (33.4)	−0.068 (0.060)	0.255	99.8 (146.1)	−0.082 (0.063)	0.191
Intermediate metaboliser	21.4 (34.0)	−0.048 (0.024)	0.045	98.7 (143.3)	−0.051 (0.025)	0.043
Normal metaboliser	22.0 (34.1)	-	-	101.8 (143.7)	-	-
Fast metaboliser	21.6 (33.3)	−0.014 (0.023)	0.531	99.7 (141.6)	−0.015 (0.024)	0.525
**Escitalopram**						
**Metaboliser phenotype**	**Count-based definition**			**Weeks-based definition**		
	**Mean (SD)**	**β (SE)**	***p***	**Mean (SD)**	**β (SE)**	***p***
Poor metaboliser	10.5 (17.6)	−0.351 (0.174)	0.043	53.3 (90.1)	−0.369 (0.185)	0.046
Intermediate metaboliser	17.1 (28.4)	−0.073 (0.067)	0.274	81.9 (130.2)	−0.079 (0.071)	0.265
Normal metaboliser	18.6 (36.3)	-	-	87.8 (134.5)	-	-
Fast metaboliser	19.1 (32.0)	0.074 (0.063)	0.244	89.1 (136.8)	0.066 (0.068)	0.326
**Sertraline**						
**Metaboliser phenotype**	**Count-based definition**			**Weeks-based definition**		
	**Mean (SD)**	**β (SE)**	***p***	**Mean (SD)**	**β (SE)**	***p***
Poor metaboliser	18.7 (35.7)	−0.123 (0.081)	0.128	82.4 (138.6)	−0.148 (0.084)	0.079
Intermediate metaboliser	18.2 (33.0)	−0.050 (0.033)	0.126	84.7 (141.9)	−0.057 (0.034)	0.095
Normal metaboliser	19.2 (37.6)	-	-	81.0 (137.9)	-	-
Fast metaboliser	17.9 (32.4)	−0.060 (0.031)	0.056	78.3 (130.5)	−0.071 (0.032)	0.029

## Data Availability

Data used in this study are available from the UK Biobank subject to standard procedures (see https://www.ukbiobank.ac.uk/ and http://biobank.ndph.ox.ac.uk/showcase/). The underlying code used to generate results will be returned to the UK Biobank, and is available upon request.

## Supplementary Materials

The following supporting information can be downloaded at: https://www.mdpi.com/article/10.3390/ph16091277/s1, Table S1: Associations between CYP2C19 metaboliser phenotype and antidepressant switching; Table S2: Associations between CYP2C19 metaboliser phenotype and antidepressant discontinuation; Table S3: Associations between CYP2C19 metaboliser phenotype and side effects following first antidepressant prescription; Table S4: Associations between CYP2C19 metaboliser phenotype and antidepressant switching in a subset of individuals that exhibited a broad depression phenotype; Table S5: Associations between CYP2C19 metaboliser phenotype and antidepressant discontinuation in a subset of individuals that exhibited a broad depression phenotype; Table S6: Associations between CYP2C19 metaboliser phenotype and antidepressant duration in a subset of individuals that exhibited a broad depression phenotype; Table S7: Associations between CYP2C19 metaboliser phenotype and side effects following initial antidepressant prescription in a subset of individuals that exhibited a broad depression phenotype; Table S8: Associations between CYP2C19 activity score and antidepressant switching; Table S9: Associations between CYP2C19 activity score and antidepressant discontinuation; Table S10: Associations between CYP2C19 activity score and antidepressant duration; Table S11: Associations between CYP2C19 activity score and side effects following initial antidepressant prescription; Table S12: Full list of read v2 and CTV3 clinical event codes pertinent to side effects for psychotropic drugs, based on the UKU side effect rating scale (UKU-SERS); Table S13: Calculation of CYP2C19 activity score based on all star alleles called in this dataset.
