# Supplementary material for: The Effects of CYP2C19 Genotype on Proxies of SSRI Antidepressant Response in the UK Biobank
Source: Pharmaceuticals (Basel). 2023 Sep 11;16(9):1277. doi: 10.3390/ph16091277 (PMC10535191; doi:10.3390/ph16091277)
Supplement: Supplementary file 1 [file pharmaceuticals-16-01277-s001.zip › pharmaceuticals-2543551-supplementary.pdf]

Supplementary Table S1: Associations between CYP2C19 metaboliser phenotype and antidepressant switching.

**Citalopram**												
**Metaboliser phenotype**	**30 days**				**60 days**				**90 days**			
	Switch frequency	% switch	OR (95% CI)	*P*	Switch frequency	% switch	OR (95% CI)	*P*	Switch frequency	% switch	OR (95% CI)	*P*
PM	17/478	3.6%	0.705 (0.428-1.160)	0.169	28/489	5.7%	0.917 (0.617-1.363)	0.669	46/506	9.1%	0.956 (0.698-1.310)	0.781
IM	233/4724	4.9%	0.981 (0.827-1.162)	0.822	287/4778	6.0%	0.964 (0.827-1.124)	0.643	488/4960	9.8%	1.04 (0.920-1.174)	0.533
NM	362/7197	5.0%	-	-	455/7290	6.2%	-	-	716/7535	9.5%	-	-
FM	300/5842	5.1%	1.019 (0.870-1.194)	0.812	367/5909	6.2%	0.991 (0.859-1.144)	0.904	582/6102	9.5%	1.005 (0.895-1.129)	0.930
**Escitalopram**												
**Metaboliser phenotype**	**30 days**				**60 days**				**90 days**			
	Switch frequency	% switch	OR (95% CI)	*P*	Switch frequency	% switch	OR (95% CI)	*P*	Switch frequency	% switch	OR (95% CI)	*P*
PM	8/54	14.8%	2.244 (0.917-5.491)	0.077	10/56	17.9%	2.311 (1.034-5.163)	0.041	13/59	22%	1.842 (0.920-3.69)	0.085
IM	36/567	6.3%	1.027 (0.650-1.620)	0.910	57/588	9.7%	1.347 (0.913-1.986)	0.133	86/613	14%	1.317 (0.955-1.817)	0.093
NM	58/866	6.7%	-	-	71/879	8.1%	-	-	106/909	11.7%	-	-
FM	30/713	4.2%	0.601 (0.374-0.966)	0.036	42/725	5.8%	0.708 (0.468-1.073)	0.103	70/749	9.3%	0.797 (0.570-1.112)	0.182
**Sertraline**												
**Metaboliser phenotype**	**30 days**				**60 days**				**90 days**			
	Switch frequency	% switch	OR (95% CI)	*P*	Switch frequency	% switch	OR (95% CI)	*P*	Switch frequency	% switch	OR (95% CI)	*P*
PM	24/238	10.1%	1.363 (0.872-2.131)	0.174	29/243	11.9%	1.331 (0.883-2.007)	0.172	41/253	16.2%	1.277 (0.898-1.817)	0.173
IM	185/2390	7.7%	1.018 (0.837-1.239)	0.856	229/2434	9.4%	1.031 (0.862-1.232)	0.740	338/2525	13.4%	1.01 (0.870-1.173)	0.897
NM	279/3633	7.7%	-	-	340/3694	9.2%	-	-	513/3841	13.4%	-	-
FM	237/2805	8.4%	1.104 (0.919-1.326)	0.291	302/2870	10.5%	1.149 (0.974-1.356)	0.100	441/2995	14.7%	1.12 (0.975-1.288)	0.109

Supplementary Table S2: Associations between CYP2C19 metaboliser phenotype and antidepressant discontinuation.

**Citalopram**								
**Metaboliser phenotype**	**Discontinuation after single prescription**				**Discontinuation after brief prescription period**			
	Discontinue frequency	% discontinue	OR (95% CI)	*P*	Discontinue frequency	% discontinue	OR (95% CI)	*P*
PM	75/536	14.0%	1.267 (0.980-1.637)	0.071	103/556	18.5%	1.366 (1.090-1.712)	0.007
IM	589/5080	11.6%	1.028 (0.920-1.150)	0.623	790/5241	15.1%	1.058 (0.959-1.169)	0.261
NM	880/7715	11.4%	-	-	1145/7933	14.4%	-	-
FM	681/6223	10.9%	0.958 (0.861-1.067)	0.437	907/6411	14.1%	0.982 (0.893-1.080)	0.704
**Esitalopram**								
**Metaboliser phenotype**	**Discontinuation after single prescription**				**Discontinuation after brief prescription period**			
	Discontinue frequency	% discontinue	OR (95% CI)	*P*	Discontinue frequency	% discontinue	OR (95% CI)	*P*
PM	3/49	6.1%	0.613 (0.178-2.105)	0.437	6/51	11.8%	0.924 (0.367-2.324)	0.866
IM	34/565	6.0%	0.676 (0.437-1.044)	0.078	52/578	9.0%	0.772 (0.536-1.112)	0.164
NM	79/887	8.9%	-	-	106/910	11.6%	-	-
FM	54/737	7.3%	0.806 (0.552-1.178)	0.266	77/758	10.2%	0.847 (0.611-1.173)	0.317
**Sertraline**								
**Metaboliser phenotype**	**Discontinuation after single prescription**				**Discontinuation after brief prescription period**			
	Discontinue frequency	% discontinue	OR (95% CI)	*P*	Discontinue frequency	% discontinue	OR (95% CI)	*P*
PM	37/251	14.7%	1.332 (0.919-1.930)	0.130	47/260	18.1%	1.210 (0.866-1.692)	0.264
IM	296/2501	11.8%	1.021 (0.870-1.197)	0.802	382/2577	14.8%	0.954 (0.828-1.098)	0.509
NM	443/3797	11.7%	-	-	612/3941	15.5%	-	-
FM	347/2915	11.9%	1.025 (0.880-1.193)	0.755	461/3010	15.3%	0.984 (0.861-1.125)	0.816

Supplementary Table S3: Associations between CYP2C19 metaboliser phenotype and side effects following first antidepressant prescription.

**Citalopram**								
**Metaboliser phenotype**	**Side effects within 30 days**				**Side effects within 60 days**			
	Side effects frequency	% side effects	OR (95% CI)	*P*	Side effects frequency	% side effects	OR (95% CI)	*P*
PM	36/675	5.3%	0.936 (0.661-1.326)	0.710	54/675	8.0%	0.892 (0.669-1.191)	0.439
IM	354/6455	5.5%	0.969 (0.844-1.113)	0.658	548/6455	8.5%	0.963 (0.860-1.078)	0.511
NM	547/9708	5.6%	-	-	851/9708	8.8%	-	-
FM	408/7833	5.2%	0.917 (0.803-1.046)	0.198	620/7833	7.9%	0.892 (0.801-0.995)	0.040
**Esitalopram**								
**Metaboliser phenotype**	**Side effects within 30 days**				**Side effects within 60 days**			
	Discontinue frequency	% discontinue	OR (95% CI)	*P*	Discontinue frequency	% discontinue	OR (95% CI)	*P*
PM	9/75	12.0%	3.179 (1.420-7.121)	0.005	10/75	13.3%	1.748 (0.836-3.656)	0.138
IM	46/791	5.8%	1.241 (0.826-1.865)	0.298	73/791	9.2%	1.082 (0.783-1.497)	0.632
NM	62/1195	5.2%	-	-	107/1195	9.0%	-	-
FM	46/946	4.9%	0.967 (0.645-1.449)	0.870	74/946	7.8%	0.853 (0.619-1.175)	0.330
**Sertraline**								
**Metaboliser phenotype**	**Side effects within 30 days**				**Side effects within 60 days**			
	Discontinue frequency	% discontinue	OR (95% CI)	*P*	Discontinue frequency	% discontinue	OR (95% CI)	*P*
PM	17/347	4.9%	0.970 (0.584-1.613)	0.908	29/347	8.4%	0.981 (0.660-1.459)	0.924
IM	177/3297	5.4%	1.032 (0.846-1.259)	0.754	292/3297	8.9%	1.013 (0.866-1.185)	0.873
NM	258/4945	5.2%	-	-	435/4945	8.8%	-	-
FM	190/3910	4.9%	0.932 (0.768-1.132)	0.480	319/3910	8.2%	0.920 (0.790-1.072)	0.285

Supplementary Table S4: Associations between CYP2C19 metaboliser phenotype and antidepressant switching in a subset of individuals that exhibited a broad depression phenotype.

**Citalopram**												
**Metaboliser phenotype**	**30 days**				**60 days**				**90 days**			
	Switch frequency	% switch	OR (95% CI)	*P*	Switch frequency	% switch	OR (95% CI)	*P*	Switch frequency	% switch	OR (95% CI)	*P*
PM	13/369	3.5%	0.699 (0.396-1.236)	0.218	19/375	5.1%	0.805 (0.500-1.296)	0.372	36/391	9.2%	0.954 (0.668-1.361)	0.794
IM	190/3848	4.9%	1.001 (0.829-1.209)	0.990	232/3890	6.0%	0.960 (0.810-1.138)	0.638	386/4031	9.6%	1.001 (0.874-1.146)	0.992
NM	294/5927	5.0%	-	-	376/6009	6.3%	-	-	595/6216	9.6%	-	-
FM	252/4787	5.3%	1.069 (0.898-1.273)	0.451	310/4845	6.4%	1.024 (0.875-1.198)	0.766	484/5000	9.7%	1.014 (0.893-1.152)	0.825
**Escitalopram**												
**Metaboliser phenotype**	**30 days**				**60 days**				**90 days**			
	Switch frequency	% switch	OR (95% CI)	*P*	Switch frequency	% switch	OR (95% CI)	*P*	Switch frequency	% switch	OR (95% CI)	*P*
PM	7/50	14.0%	2.208 (0.850-5.734)	0.104	9/52	17.3%	2.371 (1.017-5.530)	0.046	12/55	21.8%	1.959 (0.945-4.061)	0.070
IM	32/512	6.3%	1.068 (0.657-1.734)	0.791	50/530	9.4%	1.356 (0.897-2.047)	0.148	76/554	13.7%	1.318 (0.938-1.852)	0.111
NM	51/808	6.3%	-	-	64/821	7.8%	-	-	97/850	11.4%	-	-
FM	28/655	4.3%	0.653 (0.397-1.073)	0.093	40/667	6.0%	0.773 (0.503-1.189)	0.241	67/691	9.7%	0.838 (0.593-1.184)	0.316
**Sertraline**												
**Metaboliser phenotype**	**30 days**				**60 days**				**90 days**			
	Switch frequency	% switch	OR (95% CI)	*P*	Switch frequency	% switch	OR (95% CI)	*P*	Switch frequency	% switch	OR (95% CI)	*P*
PM	16/188	8.5%	1.132 (0.660-1.943)	0.652	20/192	10.4%	1.161 (0.713-1.892)	0.548	29/200	14.5%	1.125 (0.744-1.702)	0.575
IM	151/1933	7.8%	1.068 (0.858-1.33)	0.557	187/1969	9.5%	1.09 (0.893-1.331)	0.397	284/2048	13.9%	1.071 (0.908-1.263)	0.415
NM	223/2993	7.5%	-	-	269/3039	8.9%	-	-	416/3165	13.1%	-	-
FM	182/2279	8.0%	1.087 (0.883-1.337)	0.432	233/2330	10.0%	1.149 (0.953-1.386)	0.146	352/2438	14.4%	1.127 (0.965-1.317)	0.131

Supplementary Table S5: Associations between CYP2C19 metaboliser phenotype and antidepressant discontinuation in a subset of individuals that exhibited a broad depression phenotype.

**Citalopram**								
**Metaboliser phenotype**	**Discontinuation after single prescription**				**Discontinuation after brief prescription period**			
	Discontinue frequency	% discontinue	OR (95% CI)	*P*	Discontinue frequency	% discontinue	OR (95% CI)	*P*
PM	49/405	12.1%	1.446 (1.054-1.983)	0.022	62/415	14.9%	1.426 (1.073-1.896)	0.015
IM	351/4009	8.8%	1.009 (0.875-1.163)	0.905	482/4118	11.7%	1.056 (0.933-1.196)	0.389
NM	537/6170	8.7%	-	-	704/6314	11.1%	-	-
FM	382/4917	7.8%	0.890 (0.775-1.022)	0.097	514/5027	10.2%	0.916 (0.811-1.034)	0.157
**Esitalopram**								
**Metaboliser phenotype**	**Discontinuation after single prescription**				**Discontinuation after brief prescription period**			
	Discontinue frequency	% discontinue	OR (95% CI)	*P*	Discontinue frequency	% discontinue	OR (95% CI)	*P*
PM	1/44	2.3%	0.288 (0.037-2.245)	0.235	4/46	8.7%	0.838 (0.272-2.584)	0.758
IM	24/504	4.8%	0.634 (0.380-1.057)	0.081	35/512	6.8%	0.692 (0.449-1.068)	0.096
NM	59/816	7.2%	-	-	80/834	9.6%	-	-
FM	31/658	4.7%	0.612 (0.383-0.979)	0.041	46/672	6.8%	0.655 (0.441-0.973)	0.036
**Sertraline**								
**Metaboliser phenotype**	**Discontinuation after single prescription**				**Discontinuation after brief prescription period**			
	Discontinue frequency	% discontinue	OR (95% CI)	*P*	Discontinue frequency	% discontinue	OR (95% CI)	*P*
PM	17/189	9.0%	1.099 (0.649-1.858)	0.726	23/194	11.9%	1.026 (0.648-1.624)	0.914
IM	188/1970	9.5%	1.085 (0.889-1.324)	0.424	236/2016	11.7%	0.963 (0.808-1.149)	0.678
NM	272/3042	8.9%	-	-	384/3143	12.2%	-	-
FM	197/2294	8.6%	0.956 (0.786-1.163)	0.652	271/2360	11.5%	0.924 (0.780-1.094)	0.359

Supplementary Table S6: Associations between CYP2C19 metaboliser phenotype and antidepressant duration in a subset of individuals that exhibited a broad depression phenotype.

**Citalopram**						
**Metaboliser phenotype**	**Count-based definition**			**Weeks-based definition**		
	Mean (SD)	β (SE)	*P*	Mean (SD)	β (SE)	*P*
Poor metaboliser	23.2 (34.8)	-0.078 (0.069)	0.258	112.5 (157.5)	-0.076 (0.073)	0.3
Intermediate metaboliser	23.5 (35.8)	-0.055 (0.027)	0.043	109.2 (152.1)	-0.056 (0.029)	0.05
Normal metaboliser	24.4 (36.1)	-	-	113.3 (152.7)	-	-
Fast metaboliser	24.1 (35.3)	-0.009 (0.026)	0.733	112.3 (151.3)	-0.004 (0.027)	0.883
**Escitalopram**						
**Metaboliser phenotype**	**Count-based definition**			**Weeks-based definition**		
	Mean (SD)	β (SE)	*P*	Mean (SD)	β (SE)	*P*
Poor metaboliser	11.2 (18.4)	-0.377 (0.185)	0.042	57.6 (94.6)	-0.401 (0.197)	0.042
Intermediate metaboliser	18.2 (29.4)	-0.079 (0.071)	0.262	87.1 (134.1)	-0.087 (0.075)	0.245
Normal metaboliser	19.7 (37.6)	-	-	93.1 (138.1)	-	-
Fast metaboliser	20.4 (33.2)	0.074 (0.067)	0.273	95.1 (141.6)	0.061 (0.071)	0.396
**Sertraline**						
**Metaboliser phenotype**	**Count-based definition**			**Weeks-based definition**		
	Mean (SD)	β (SE)	*P*	Mean (SD)	β (SE)	*P*
Poor metaboliser	21.7 (39.6)	-0.077 (0.095)	0.419	94.8 (151.2)	-0.102 (0.099)	0.301
Intermediate metaboliser	20.3 (35.8)	-0.072 (0.037)	0.055	90.6 (149.0)	-0.078 (0.039)	0.044
Normal metaboliser	21.5 (40.6)	-	-	95.3 (153.5)	-	-
Fast metaboliser	19.6 (34.6)	-0.071 (0.036)	0.045	86.9 (140.3)	-0.082 (0.037)	0.026

Supplementary Table S7: Associations between CYP2C19 metaboliser phenotype and side effects following initial antidepressant prescription in a subset of individuals that exhibited a broad depression phenotype.

**Citalopram**								
**Metaboliser phenotype**	**Side effects within 30 days**				**Side effects within 60 days**			
	Side effects frequency	% side effects	OR (95% CI)	*P*	Side effects frequency	% side effects	OR (95% CI)	*P*
PM	26/501	5.2%	0.888 (0.59-1.335)	0.567	38/501	7.6%	0.828 (0.588-1.165)	0.278
IM	284/5058	5.6%	0.967 (0.829-1.129)	0.673	441/5058	8.7%	0.97 (0.855-1.101)	0.639
NM	444/7685	5.8%	-	-	686/7685	8.9%	-	-
FM	327/6138	5.3%	0.913 (0.788-1.059)	0.229	489/6138	8.0%	0.881 (0.780-0.995)	0.041
**Esitalopram**								
**Metaboliser phenotype**	**Side effects within 30 days**				**Side effects within 60 days**			
	Discontinue frequency	% discontinue	OR (95% CI)	*P*	Discontinue frequency	% discontinue	OR (95% CI)	*P*
PM	5/66	7.6%	1.737 (0.629-4.798)	0.287	6/66	9.1%	1.033 (0.415-2.57)	0.945
IM	43/696	6.2%	1.342 (0.875-2.059)	0.177	65/696	9.3%	1.058 (0.751-1.49)	0.748
NM	55/1077	5.1%	-	-	99/1077	9.2%	-	-
FM	45/841	5.4%	1.063 (0.698-1.619)	0.775	70/841	8.3%	0.863 (0.618-1.204)	0.386
**Sertraline**								
**Metaboliser phenotype**	**Side effects within 30 days**				**Side effects within 60 days**			
	Discontinue frequency	% discontinue	OR (95% CI)	*P*	Discontinue frequency	% discontinue	OR (95% CI)	*P*
PM	12/260	4.6%	0.893 (0.488-1.633)	0.713	20/260	7.7%	0.875 (0.544-1.407)	0.581
IM	144/2595	5.5%	1.067 (0.854-1.332)	0.569	238/2595	9.2%	1.033 (0.867-1.23)	0.715
NM	205/3942	5.2%	-	-	352/3942	8.9%	-	-
FM	150/3075	4.9%	0.944 (0.758-1.174)	0.603	257/3075	8.4%	0.938 (0.791-1.112)	0.461

Supplementary Table S8: Associations between CYP2C19 activity score and antidepressant switching.

**Antidepressant switching at 3 timepoints**						
**SSRI**	**30 days**		**60 days**		**90 days**	
	B (SE)	*P*	B (SE)	*P*	B (SE)	*P*
Citalopram	0.054 (0.04)	0.172	0.027 (0.036)	0.454	0.009 (0.029)	0.763
Escitalopram	-0.311 (0.11)	0.005	-0.337 (0.096)	<0.001	-0.249 (0.079)	0.002
Sertraline	0.027 (0.046)	0.559	0.039 (0.041)	0.34	0.041 (0.035)	0.235

Supplementary Table S9: Associations between CYP2C19 activity score and antidepressant discontinuation.

**Antidepressant discontinuation**				
**SSRI**	**Discontinuation after single prescription**		**Discontinuation after brief prescription period**	
	B (SE)	*P*	B (SE)	*P*
Citalopram	-0.041 (0.026)	0.114	-0.051 (0.023)	0.029
Escitalopram	0.003 (0.098)	0.972	-0.064 (0.083)	0.446
Sertraline	-0.009 (0.038)	0.821	-0.006 (0.033)	0.846

Supplementary Table S10: Associations between CYP2C19 activity score and antidepressant duration.

Antidepressant duration				
**SSRI**	**Count-based definition**		**Weeks-based definition**	
	B (SE)	*P*	B (SE)	*P*
Citalopram	0.009 (0.011)	0.397	0.010 (0.012)	0.385
Escitalopram	0.072 (0.031)	0.021	0.068 (0.031)	0.030
Sertraline	0.071 (0.033)	0.033	0.067 (0.033)	0.044

Supplementary Table S11: Associations between CYP2C19 activity score and side effects following initial antidepressant prescription.

Side effects following initial antidepressant prescription within 3 timepoints						
**SSRI**	**30 days**		**60 days**		**90 days**	
	B (SE)	*P*	B (SE)	*P*	B (SE)	*P*
Citalopram	-0.001 (0.033)	0.974	-0.015 (0.027)	0.58	-0.018 (0.024)	0.465
Escitalopram	-0.194 (0.100)	0.052	-0.170 (0.079)	0.032	-0.157 (0.070)	0.026
Sertraline	-0.043 (0.047)	0.364	-0.049 (0.037)	0.186	-0.046 (0.033)	0.172

Supplementary Table S12: Full list of read v2 and CTV3 clinical event codes pertinent to side effects for psychotropic drugs, based on the UKU side effect rating scale (UKU-SERS).

UKU-SERS Item	Corresponding read v2 and CTV3 codes
Concentration difficulties	1BR..; 1BR0.; 1BW..; 8B39C; Ua1XX; X760c; X760d; .1BR.; .1BR0; .1BW.
Asthenia/Lassitude/ Increased Fatiguability	Eu460; R0072; R007z; 168..; 1682; R007.; R0071; 1683; 168Z.; E205.; R0075; E2622; Eu453; XM0yx; XM1AV; .E36.; XE0uN; XM06l; XM0D3; .168.; .1682; .R07.; .R071; .R07Z; XM06o; .1683; .168Z
Sleepiness/sedation	R0053; R00z4; X007z; X0080; X0082; X76AR; XE2nU; XM06R; Xa2bY; .R053; XM0yz; X769V
Failing memory	1B1a.; 1B1A.; 1B1A0; 1B1A1; 1B1Y.; 1S21.; 1S23.; 3A10.; 3A20.; 3A30.; 3A40.; 3A50.; 3A60.; 3A70.; 3A80.; 3A91.; 3AA1.; 3AG1.; E2A10; R00z0; 28E..; 28E0.; 28E1.; 28E2.; 28E3.; 3AE1.; 3AE2.; 3AE3.; 3AE4.; 3AE5.; 3AE6.; Eu057; Ua196; Ua197; X75xC; X75xD; X75xG; X75xH; X75xU; X75xY; XE1bq; Xa2Ve; XaB5o; Ua189; X00RS; XaJBS; XaJBT; XaJBU; XaJBV; XaJBW; XaJBX; Xaagi; Xaagj; XaJfj; .1B1A; .1B1Y; .1B1a; .1S21; .3A10; .3A20; .3A30; .3A4 0; .3A50; .3A60; .3A70; .3A80; .3A91; .3AA1; .E4J3; .R0Z0; Xaagk; .28E.; .3AE1; .3AE2; .3AE3; .3AE4; .3AE5; .3AE6; .49 B4; .E16.
Depression	1B17.; 1B1U.; 1BT..; 2257.; E02y3; E112.; E1120; E1121; E1122; E1123; E1124; E112z; E113.; E1130; E1131; E1132; E1133; E1134; E1137; E113z; E11y2; E11z2; E135.; E130.; Eu32.; Eu320; Eu321; Eu322; Eu323; Eu324; Eu325; Eu326; Eu327; Eu328; Eu329; Eu32A; Eu32y; Eu32z; Eu33.; Eu330; Eu331; Eu332; Eu333; Eu33y; Eu33z; Eu3z.; E1150; E1151; E1152; E1153; E1154; E1155; E1156; E115z; E11y0; E2003; Eu412; Eu3y1; E11..; E1125; E1126; E1135; E1136; E114.; E115.; E11y.; E11y3; E11yz; Eu313; Eu314; X00SM; X00SO; X00SQ; X00SR; X00SU; X00Sb; XE0re; XE0uv; XE1Y0; XE1Y1; XE1ZY; XE1ZZ; XE1Za; XE1Zb; XE1Zc; XE1Zd; XE1Ze; XE1Zf; XE1aQ; XE1aS; XM0CR; XM1GC; XSEGJ; XSGok; XSGol; XSGom; XSGon; Xa0wV; XaB95; XaB9J; XaCHo; XaCHr; XaCHs; XaCIs; XaCIt; XaCIu; XaImU; .1B17; .1B1U; .1BT.; .2257; .E22.; .E221; .E222; .E22Z ; .E478; .E4J5; .E4J6; .E4J7; .E4J8; .E4J9; .E4JC; Eu204; Eu251; Eu31.; Eu315; Eu341; X00SH; Xa0Rd
Tension/Inner unrest	1B14.; 1B12.; R2y2.; 1B1O.; R00zD; 1B16.; 2256.; XC0CP; .1B14; .1B12; XE0ra; .RJ2.; XM1Af; .1B1O; Ua15v; XE0rd; .1B16; .2256
Increased duration of sleep	1BX1.; 1B1Q.; R0056; R0057; X007w; X0080; X76AQ; .1BX1; .1B1Q; X76AE; XaFqr; XM06j
Reduced duration of sleep	1B1B.; 1B1Q.; E274D; R005.; R0051; E274.; E274E.; E274B.; R0058; R0059; R005z; R0056; R0057; E274B; E274E; X007v; X76AG; X76AN; XE0ux; XE1Yi; XE2Pv; XE2cd; XM06i; Xa7wV; .1B1B; .R05.; .R05Z; XM06j; XaFqr; .1B1Q; R0056.; X76AE

Increased dream activity	1BX4.; 1BX5.; 1BX6.; 1BX7.; 1BX8.; E274y; Ua1ZR; X764A; X764B; X764C; XaKSR; XaKSS; XaKST; XaKSU; XaKSV; .1BX4; .1BX5; .1BX6; .1BX7; .1BX8
Emotional indifference	2254.; R00z7; XE1ik; XM013; .2254; X760R; X80ws; Xa3HP; Ua1r0; Ua1r1
Dystonia	7Q040; F136.; F1372; F138.; F138z; F13A.; F13B.; F13X.; Fyu24; Fyu2A; Ub1TW; X0049; X004F; X004G; X004H; X004I; X004M; X004N; X004Q; X004R; X004T; X004X; X76qI; XaMEg; XaNms; .F235; F13C.
Rigidity	1B35.; 25F..; 25F2.; 25FZ.; 294..; 2943.; 2944.; R094.; 2942.; X76gL; X76qN; XM03C; XM03z; XM040; XM098; XM1Wv; XaJgO; .25F.; .25F2; .25FZ; .294.; .2943; .2944; F13z3; .R94.; .M683; XaE6P; .1B35; XE1hV; XE1jG; X76qO
Hypokinesia/akinesia	F13z3; 1P01.; 1P03.; .1P01; .1P03; X763E
Hyperkinesia	E2E1.; E2E2.; E2Ey.; E2Ez.; Eu90.; Eu90y; Eu90z; J105.; J65yB; F1381; R013.; E2E..; X004Z; X004a; X004b; X004c; X78x7; XE0aN; XE15N; XE17n; .E4H.; .F234
Tremor	1B22.; 2975.; 2976.; 2977.; 297B.; 297C.; E2013; F1312; Fyu25; R0103; 1B23.; X004l; X004m; X004n; X004o; X004p; X76ns; X76nt; X76nv; XE0rn; XE1jk; XM047; XM048; XM0z1; XaKV5; XaKV6; .1B22; .2975; .2976; .2977; .R103; XE0ro; XM1F4; .1B23
Akathisia	1P04.; X004d; Xa306; XaKVg; .1P04; .F369
Epileptic seizures	282..; F2516; F25H.; R0034; R003z; F25C.; F253.; F25X.; F25z.; Fyu50; Fyu59; X006M; X75YS; X75Z0; XM03h; XM06d; Xa0Ji; XaBM2; XaEHz; XaEIj; XaREJ; .282.; .R03Z; F2510; Fyu52; X006t; X007B; .F342; .F343; .F347
Paraesthesia	1B46.; 1B47.; 29B5.; 29B50; F351.; G73y4; G73y5; G73y6; R0207; 1B43.; R0203; 1B44.; 1B442; R0206; 2G2D.; 1B42.; R0202; R0201; X205S; XM07I; Xa0We; XaBj9; XaBjA; XaBtl; XaCIS; XaINq; .1B46; .1B47; .29B5; .F441; .R207; X78xq; XE0v1; XM1BF; .1B43; .R203; XE0v3; XM07H; Xa4XI; Xa6mx; Xa71Y; Xa7QR; XaBtR; XaBtg; XaBth; XaBti; XaBtj; XaBu3; XaCIy; .1B44; .R206; XM1BB; Xa485; .R201; XE0uz; .1B42; .R202
Accommodation disturbance	2BI6.; F47..; F475.; F4750; F4752; F475z; F47y.; F47yz; F47z.; FyuK.; F4816; X00fN; X00fO; X00fP; X00gD; X77OP; X77OQ; XE18t; .2BI6; .F56.; .F565; .F56Z; X78x2
Increased salivation	1925.; 2532.; R124.; XE0r6; XM1Mv; .1925; .2532
Reduced salivation	J077.; J0770; J077z; 1927.; Ryu78; X76bF; Xa7TC; Xa7TE; X76bE; .1927
Nausea/vomiting	198..; 1982.; 198Z.; R070.; R0700; R070z; R0701; R0703; 199..; 1992.; 1993.; 199Z.; 19FZ.; 19G..; J162.; X75qw; Xa1pJ; Xa7ee; .198.; .1982; .198Z; .R70.; .R700; .R70Z; XM0Cl; X76co; XE0rA; XM0Cm; XaBMW; .199.; .1992; .1993; .199Z; .R701; R0704; .19FZ; .19G.
Diarrohea	19F..; 19F2.; 19F3.; J4...; J4z..; E2643; 19G..; 4743.; 4744.; 19EE.; 2AF3.; J432.; J4zz.; J5201; X30Bp; X76dE; XE0rN; XE0rO; XM1Mz; .19F.; .19F2; .19F3; .19G.; J525.; .4743;

	.4744; Xa7VE; .19EE; .2AF3; X30Bq
Constipation	19C..; 19C2.; E2645; J520.; J5200; J5203; J520z; J520y; J50zz; J52y1; 2AF2.; X30Bm; XE0cn; XE0rD; Xa7n2; XaE1r; .19C.; .19C2; .2AF2; .I63.; X305B; Xa1hS; Xa1hT; .I61.; .4745; 4745; Xa7VB
Micturition disturbances	1A33.; 1A34.; 1AZ3.; R086.; Ryu40; 1A32.; R082.; R0822; R0823; R0824; R0860; R0863; .1A33; .1A34; .1AZ3; .R86.; 1AC0.; 1AC1.; Ua1sm; X76Xb; X76Xp; X76Xt; X76Xx; X76Xz; XE0rS; .1A32; .1AC1; XaD2w; .R82.; .R820; .R821; .R822; X30O4
Polyuria/polydipsia	1A1..; 1A12.; 1A1Z.; 1A25.; R084.; R0840; R084z; R0862; 1A26.; 1A27.; 1AC2.; R0841; 1644.; 1645.; R035.; R035z; R0350; .1A1.; .1A12; .1A1Z; .1A25; .1A35; .R84.; .R84Z; 1A35.; X76Xs; X76XV; XE0rQ; XE2RD; XM1CC; .1A27; .1AC2; X30OG; X76Xr; Xa7Wt; XaNFc; .R840; XE0qd; .1644; .R33.; .R33Z; X76cR; .R330
Orthostatic dizziness	G870.; 1B55.; 1B5..; 1B53.; R0040; X00CT; XaNXF; .G971; XaJDf; .1B55; XC07f; XE0v5; XM06f; XM06g; .1B5.; .1B53; .R040
Palpitations	181..; 1812.; 181Z.; R051.; R051z; 1814.; 1813.; X76Jb; X76Je; X76Jo; XE0qv; .181.; .1812; .1813; .1814; .181Z; .R51.; .R51Z
Increased tendency to sweating	166..; 1662.; 2223.; R0081; R0084; R008.; R0082; R0083; R008z; 166Z.; X76A3; X76A4; X76A5; XE0qg; XM06r; Xa7ls; .166.; .1662; .166Z; .2223; XM1BD; XaDml; .R08.; .R08Z
Rash	1D14.; 2227.; 2I14.; M130.; R021.; R021z; 2536.; 2FR..; 2FU..; 2FW0.; 2I1B.; 2I1C.; M2y42; M28..; M280.; M281.; M28z.; Myu4.; 2F23.; 2FS..; R0270; R0210; M28y.; X50Ge; X75uU; X75uX; XE0vP; XE1gz; XM07J; XM07T; XM1YL; Xa7yK; Xa9wq; XaB3a; XaBsZ; XaEEr; XaIO6; XaIq5; XaIwz; XaIx0; .1D14; .2227; .2FR.; .2FS.; .2FU.; .2FW0; .2I14; .2I1B; .2I1C; .L49.; .R21.; .R212; .R21Z; XE2cX; XM11k; .2F23; .R270; X508N; XE1BT; XE1D0; XE1tH; Xa8EW; .L491; .L49Z; .P9G2; X50C5; .R210
Pruritus	M180.; M18..; Myu2D; 1D15.; M18z.; Myu2B; XE1B7; XE1BA; XE1CY; Xa0Wb; XM00q; Xa05c; Xa13L; .L351; .L35.; .L35Z; XE0sH; .1D15; XM1Xn
Photosensitivity	M1274; F25F.; M1273; X504r; X505T; X50Gr; XE1CC; .L247; X006y; X75b9
Increased pigmentation	2275.; M292.; 2FM1.; X75tt; XE1D2; Xa6mT; .L4A.; .L4A3; XaIuv; .2FM1
Weight gain	1622.; 1624.; 1629.; R031.; Ua16s; X76C9; X90kO; Xaazc; .1622; .1624; .R301
Weight loss	1623.; 1625.; 1627.; 1D1A.; R032.; 22A8.; X76CA; X90kN; XE0qb; XE0uH; XaBmk; XaIxC; XaKwR; XaQgK; XaXTs; .1623; .1625; .1D1A; .22A8; .R302
Menorrhagia	K5920; K593.; K5A0.; K5A6.; K598.; K592.; K5921; K596.; X408g; X408h; XE0ew; XE0h5; Xa9CP; XaFC2; .J72.; .J771; X408j; XE0eo; XE0gt; .J743; XE0er; XE0h1; .J75.

Amenorrhea	K590.; K5901; K590z; K5911; K5913; K5910; X408Z; XE0em; .J71.; .J712; .J71Z; XE0gp; XE0gr; .J741; .J742
Galactorrhoea	K316.; 26D3.; X40Nd; XM0B1; .J4Z3; .26D3
Gynaecomastia	26B2.; K3110; K3112; K311.; K311z; X40Ff; XE0eN; XE0g5; XaNz7; XaYQT; .26B2; .J4Z1
Increased sexual desire	Eu52.; Eu527; Eu52z; ZV417; E22y5; E22y6; Ua1b5; X76M4; XE1Zu; X00T2; X766y
Diminished sexual desire	1ABG.; E2271; Eu520; Eu521; E227.; E2270; E227z; Eu52.; Eu527; Eu52y; Eu52z; ZV417; Eu522; X76M2; X76M3; XE1YO; XE1YP; XE2bA; X00T2; X766x; XE1as; XaBj1; .E422; X76Ly; .1ABG
Erectile dysfunction	1D1B.; E2273; Eu522; 1ABB.; 1ABC.; Eu52.; Eu52z; ZV417; X76ME; X76MM; X76MO; Xa044; XaXgw; .1ABB; .1ABC; .J3ZB; X00T2
Ejaculatory Dysfunction	E2276; Eu524; Eu52.; Eu52z; ZV417; X30OT; X76MS; X76MU; XE1as; gm...; .E422; X76MX; X00T2
Orgastic Dysfunction	E2274; E2275; Eu523; E2272; Eu520; Eu52.; Eu52y; Eu52z; ZV417; E2276; X401t; X76MU; X76Ma; X76Mb; X76Me; X76Mf; X76Mg; X76Mh; X76Mi; XE1Zt; Eu524; XE1as; .E422; X00T2
Dry Vagina	1AD..; K5A30; K5A3.; K5A5.; X407z; X76M5; .1AD.; Xa6Yg
Headache	1B1G.; 1B1G0; 1BA2.; 1BA3.; 1BA4.; 1BA5.; 1BA6.; 1BA7.; 1BA8.; 1BA9.; 1BAZ.; 1BB1.; 1BB2.; 1BB3.; 1BB4.; 1BBZ.; E2781; F2611; F2620; F2626; F2629; F262A; F2W..; F2W0.; F2X..; Fyu54; Fyu5A; Fyu5B; R040.; F262E; X007I; X007U; X007W; X007b; X007h; XE0rh; XE0s6; XE187; XE1Yl; XM0CV; XaMIU; XaXpZ; XaXsF; .1B1G; .1BA2; .1BA3; .1BA4; .1BA5; .1BA6; .1BA7; .1BA8; .1BA9; .1BB1; .1BB2; .1BB3; .1BBZ; .E4C1; .F35Z; .F39.; .R40.; F2621; F2625; XaXsc
Physical Dependence	E24..; E241.; E2410; E241z; E247.; E2470; E247z; E24z.; 13cG.; 13cQ.; 13cR.; 13cS.; X00Rt; X00S4; XE1YS; XE1YW; .E44.; .E44Z; Ub0nr; Ub0ns; Ub0nt; .13cG; .13cR
Psychic Dependence	13cS.; 13cQ.; Ub0ns; Ub0nu; .13cQ; .13cS

Supplementary Table S13: Calculation of CYP2C19 activity score based on all star alleles called in this dataset.

Allele	Score
*1, *11, *13, *15	1
*2, *3, *4, *8, *22, *35	0
*9, *10	0.5
*17	2
*30, *34	N/A
